# Immunobiology of visceral leishmaniasis

**DOI:** 10.3389/fimmu.2012.00251

**Published:** 2012-08-14

**Authors:** Rajiv Kumar, Susanne Nylén

**Affiliations:** ^1^Department of Medicine, Institute of Medical Sciences, Banaras Hindu UniversityVaranasi, India; ^2^Department of Microbiology, Tumor and Cell Biology, Karolinska InstitutetStockholm, Sweden

**Keywords:** visceral leishmaniasis, immune regulation, *Leishmania donovani*, IL-10, T cells

## Abstract

Visceral leishmaniasis (VL), commonly known as kala-azar, is caused by *Leishmania donovani* and *Leishmania infantum* (*Leishmania chagasi* in the Americas). These *Leishmania* species infect macrophages throughout the viscera, and parasites are typically found in the spleen, liver, and bone marrow. Patients with active disease typically exhibit marked immunosuppression, lack reactivity to the *Leishmania* skin test (LST), a delayed type hypersensitivity test, and their peripheral blood mononuclear cells (PBMC) fail to respond when stimulated with leishmanial antigens *in vitro*. However, most people infected with visceralizing species of *Leishmania* never develop disease. Understanding immune failure and the underlying immune mechanism that lead to disease as well as control of infection are key questions for research in this field. In this review, we discuss immunological events described in human and experimental VL and how these can affect the outcome of infection.

## VISCERAL LEISHMANIASIS

Visceral leishmaniasis (VL) is a vector borne disease transmitted by sandflies (of the genus *Phlebotomus* in Africa, Asia, and Europe and the genus *Lutzomyia* in the Americas). Unlike most human pathogenic *Leishmania* species, which reside in macrophages of the skin and skin draining lymph nodes, *Leishmania donovani* and *Leishmania infantum* spread systemically to propagate in macrophage of internal organs, primarily the liver, the spleen, the bone marrow, and the lymph nodes.

Clinical presentation of VL typically involves long-term low-grade fever, enlarged spleen and liver and weight loss, pancytopenia and polyclonal (IgG and IgM) hypergammaglobulinemia (Badaro et al., 1986). Hypoalbuminemia seen in VL is associated with edema and other features of malnutrition. Diarrhea may occur as a result of intestinal parasitization and ulceration. Liver function may be normal or altered and in later stages of disease pro-thrombin production decreases. With time, untreated VL can cause severe cachexia and bleeding due to thrombocytopenia. The loss of thrombocytes as well as the decline in pro-thrombin can results in severe mucosal hemorrhage that may facilitate sepsis. Furthermore, loss of leucocytes eventually makes VL patients generally immunosuppressed, and bacterial infections are a common cause of death in lethal cases of VL. Untreated VL will in most cases ultimately lead to death.

The gold standard for diagnosis of all leishmaniases is demonstration of parasite (amastigote) in a tissue biopsy (Sundar and Rai, 2002). Serological tests, such as the rk39 test (Sundar et al., 2002b), are indicative of VL in combination with clinical symptoms, but cannot reliably differentiate between past and recent infections.

Fortunately, most cases of VL are treatable. The drugs used most commonly are pentavalent antimonials (SbV), Miltefosine and Amphotericin B, all expensive and associated with toxicity. In India resistance to SbV is wide spread (Agrawal et al., 2005) and reports of drug resistance to other drugs, including Amphotericin B, has been reported (Srivastava et al., 2011). At present, there is no anti-leishmanial vaccine. Thus, there is an urgent need for development of new therapeutic strategies.

Following therapeutic cure of VL**caused by* L. donovani*, patients can develop a sequel known as post kala-azar dermal leishmaniasis (PKDL; reviewed in Ganguly et al., 2010). There are no experimental models for this disease manifestation and why PKDL appear is not clear. PKDL presents as appearance of nodules, papules, and hypopigmented macules on the skin, in which *L. donovani*-infected macrophage can be found (Ramesh and Mukherjee, 1995; Zijlstra et al., 2003). In Africa, where PKDL is more common, most of cases of PKDL heal spontaneously, whereas in India self-cure is rare, if ever occurring (Zijlstra et al., 2003). In line with observations of human VL (discussed below), PKDL is associated with high level of interleukin (IL)-10 in blood and skin during clinical disease (Gasim et al., 1998).

## MODELS OF VL

Dogs, being natural host of *L. infantum* are highly relevant animal models for VL (Alvar et al., 2004). Notably, most *L. donovani* complex infections are subclinical in both canids and humans, but infection can in both species result in severe life-threatening visceral disease and many features of VL are shared (Alvar et al., 2004), while others, like the keratitis and skin pathology frequently observed in canine VL is not seen in human disease (Baneth et al., 2008). Due to high costs and ethical concerns dogs are only used to a limited extent in experimental VL, small rodent models are usually preferred.

Mouse models have been used extensively to study both *L. donovani* and *L. infantum* infection. The outcome of murine VL infection has a clear genetic basis; genetically resistant mouse (e.g., CBA) has functional *Slc11a1* gene that encodes a phagosomal component, solute carrier 11a1 also know as Nramp1, a proton/bivalent cation antiporter that localizes to late endosomes/lysosomes and confers the ability to control the early infection (Crocker et al., 1984; Blackwell et al., 1989). Susceptible strains (BALB/c and C57Bl/6) have mutant *Slc11a1* gene, which allows rapid parasite replication in the liver during the first weeks of infection (Crocker et al., 1984; Vidal et al., 1995). However, most susceptible mouse strains including BALB/c acquire immune responses that control parasite growth at later stage of infection. In these strains the rate of resolution of disease is determined by MHC class II haplotypes (H-2 loci; Blackwell, 1983).

Considering that “susceptible” mice control the disease, it may be more appropriate to view the murine VL as a model for sub-clinical or self-limiting infection rather than a model of disseminated visceral disease and parallels to overt human VL disease should, thus, be made with caution.

In mice, immune response to *L. donovani* or *L. infantum* infection can, within the same animal, vary markedly between different organs (i.e., liver and spleen). Following intravenous or intracardial infection (primarily using amastigotes), the parasites multiply rapidly in the liver for the first few weeks after which, the parasite growth is controlled by the cell-mediated immune response and granuloma formation. Over a period of 2–3 months *L. donovani*-infected mice eventually clear the parasites from the liver and become resistant to reinfection (Murray et al., 1987). While overt pathology is limited, the parasites persist in the spleen and the infection slowly but continuously progress for a longer period in the spleen as compared to the liver infection. Eventually, splenic replication is also controlled and parasites are maintained at a constant levels (Engwerda et al., 2004). In the spleen the persistent parasites cause splenomegaly and remodeling of splenic architecture with atrophy of lymphoid follicles (Engwerda et al., 2004).

Similar to observations made in humans (see below) the immune response in the murine spleen is characterized by a mixed regulatory and inflammatory response. Both IL-10 and tumor necrosis factor (TNF)α production is elevated in the spleen. TNFα, which is critical for development of protective immunity in the liver (Murray et al., 2000), cause destruction of the marginal zone macrophages and the gp38^+^ stromal cells, a subset of stromal cells confined to the T cell zone of the mouse spleen. (Carrión, 2006; Stanley and Engwerda, 2007). IL-10, which has been suggested to be induced by high levels of TNFα (Ato et al., 2002), may serve to control the tissue damage caused by TNFα, but the at the same time IL-10 promotes parasite persistence by inhibiting macrophage activation (Belkaid et al., 2001). IL-10 is a regulatory cytokine, presumed to be induced as a part of homeostatic network, to protect tissue from collateral damage caused by excessive inflammation. IL-10 has primarily suppressive effects on immune function, targeting multiple activation, and antigen presentation pathways of macrophages and dendritic cells. IL-10 renders macrophages unresponsive to activation signals and acts on dendritic cells (DCs) causing them to down-regulate CCR7 and thereby loosing migratory capacity preventing them from proper accessing T cell areas and effectively priming T cells responses (Ato et al., 2002). It has clearly been demonstrated in mouse models that IL-10 promotes diseases progression and that inhibition of IL-10 activity (through IL-10 receptor blockade) leads to more rapid granuloma formation and parasite killing (Murray et al., 2002). Blocking the IL-10R resulted in increased IFNγ production and enhanced expression of inducible nitric oxide synthase (iNOS) in infected tissue (Murray et al., 2003b). Given in combination with anti-leishmanial therapy (SbV), blockade of IL-10R was shown to enhance effectiveness of SbV (Murray et al., 2005).

Hamsters, similar to humans and canids, can develop progressive fatal disease in which parasites replicate in the liver, spleen, and bone marrow eventually causing death of the host (Ghosh and Ghosh, 1987; Requena et al., 2000; Melby et al., 2001). As in humans, hamsters have up-regulated expression of Th1-associated cytokine mRNA (IFNγ, IL-2, and TNFα in the spleen, but limited induction of IL-4 mRNA (Melby et al., 2001). Substantial amounts of TGFβ and IL-10 mRNAs are also present, which may promote parasite multiplication and survival. Similar to humans, hamsters are poor producers of nitric oxide (NO). In *L. donovani*-infected hamsters the expression of nitric oxide synthase (NOS) 2 mRNA, the gene encoding iNOS in response to IFNγ is not upregulated. This has been explained by reduced NOS2 promotor activity in hamster as compared to mice (Perez et al., 2006). The failure to induce iNOS can explain the defect in parasite killing seen in hamsters (Melby et al., 2001; Wilson et al., 2005). In contrast to humans, hamsters develop severe ascites and glomerulonephritis associated with deposition of parasite antigen immune complexes in the kidneys. The disseminated amyloidosis and glomerulonephritis results in renal failure and nephritic syndrome which is causative of death in infected hamsters (Sartori et al., 1992). Death due to renal failure is a rare event in humans and dogs.

The lack of reagents for immunological analysis and the animal’s high innate susceptibility make hamster a less suitable model for the evaluation of immune responses, but hamsters remain an efficient and a highly relevant model to test existing and experimental drugs against VL.

## IMMUNE RESPONSES IN HUMAN VL

Manifestations of human VL range from fatal visceral disease to asymptomatic infection (defined as presence of *Leishmania*-specific antibodies or being positive skin test to *Leishmania* antigen without any symptoms of VL). Whether an infection remains asymptomatic or progressed toward VL is the results of the interactions between the environment, the parasite and the host genetics, however, what more precisely determined if a person develops VL is at large still unclear.

Peripheral blood mononuclear cells (PBMCs) from some (but not all) individuals with subclinical or asymptomatic infection respond to stimulation with leishmanial antigen (LAg) and produce IL-2, IFNγ, and IL-12 (Carvalho et al., 1992). Neutralizing antibodies to IL-12 abrogates both proliferation and IFNγ production in these naturally exposed healthy individuals (Ghalib et al., 1995).

Peripheral blood mononuclear cells from active VL patients typically do not proliferate or produce IFNγ in response to LAg (Sacks et al., 1987; White et al., 1992) and most patients with active disease have a negative leishmanin skin test (LST; Manson-Bahr, 1961; Gidwani et al., 2009). A few months after completion of therapy, following cure, proliferative and cytokine responses to *Leishmania* antigen are usually detectable (White et al., 1992). The ability of PBMCs from cured VL patients to proliferate to *Leishmania* antigen can be suppressed by co-culture with PBMCs from the same patient collected prior to cure, suggesting there are immunosuppressive factors produced by these PBMCs (Carvalho et al., 1989). Interestingly, recent studies using whole blood (instead of purified PBMCs) from VL patient have shown that the blood cells maintain to capacity to produce IFNγ in response to soluble *Leishmania* antigen (Ansari et al., 2011; Gidwani et al., 2011a).

Although, VL initially was thought to be associated with a Th2-type immune response seen as elevated levels of IL-4 and/or IL-13 (Sundar et al., 1997; Nylen et al., 2007) most studies implicate that there is not a clear Th2 skewing in human VL. Typically VL is associated with increased production of multiple and primarily pro-inflammatory, cytokines and chemokines. VL patients have been found to have elevated plasma protein levels of IL-1, IL-6, IL-8, IL-12, IL-15, IFNγ inducible protein-10 (IP-10), monokine induced by IFNγ (MIG), IFNγ, and TNFα (Ansari et al., 2006; Kurkjian et al., 2006; Nylen et al., 2007). Elevated levels of IFNγ mRNA has been found in the spleen and bone marrow during the acute phase of infection (Nylen and Sacks, 2007). These observations suggest that development of VL is not driven by Th2 skewing *per se*, but that other mechanisms contribute to the pathogenesis of VL.

In support of experimental findings, clinical studies strongly implicates IL-10 in many of the immunologic defects associated with kala-azar (reviewed in Nylen and Sacks, 2007). Patients with active VL have elevated levels of IL-10 in serum as well as enhanced IL-10 mRNA levels in spleen, lymph nodes, and bone marrow (Nylen et al., 2007; Ansari et al., 2011). While antigen driven IL-10 production cannot be measured in antigen-stimulated PBMCs from VL patients, IL-10 can be detected in whole blood cells cultures of VL patients following stimulation with *Leishmania* antigen (Ansari et al., 2011). Further studies are needed to explain the difference in the ability of whole blood and PBMC to respond with IL-10 production when re-stimulated *in vitro*.

The main disease-promoting activity of IL-10 in VL is probably conditioning host macrophages for enhanced survival and growth of the parasite. IL-10 can render macrophages unresponsive to activation signals and inhibit killing of amastigotes by down-regulating the production of TNFα and NO. In human, VL inhibition of IL-10 enhance the IFNγ response by antigen-stimulated PBMC, and neutralization of IL-10 in VL serum inhibit *L. donovani* replication in macrophages (Ghalib et al., 1995; Nylen et al., 2007). A recent study from India has directly demonstrated an anti-parasitic effect of IL-10 blockade in splenic aspirates from patients with VL (Gautam et al., 2011). Taken together, the data strongly support a role for IL-10 in promoting pathology of VL and suggest that targeting IL-10 may be a method to improve VL therapy and disease outcome as discussed below.

Another cytokine implicated in the pathology of both experimental and human leishmanial disease progression is TGFβ (Barral-Netto et al., 1992; Saha et al., 2007). TGFβ has down-modulatory effects on macrophages and its blockade has been found to limit parasite replication in these cells (Reed, 1999; Anderson et al., 2008). The role of TGFβ in VL is not confirmed, but anticipated to act in synergy with IL-10 as suggested for murine cutaneous leishmaniasis (mCL).

The majority of individuals infected with *L. donovani* or *L. infantum* never develop disease, suggesting that genetic factors may be involved in resistance and susceptibility. Polymorphism in *SLC11a/NRAMP* gene, which is linked to murine resistance, is not clearly linked to development of disease in humans; while studies of Sudanese populations have found an association of *SLC11a* gene in regulating susceptibility with human VL (Bucheton et al., 2003; Mohamed et al., 2004), a more recent study made in an Indian population showed no evidence of such association (Mehrotra et al., 2011).

Notably, environmental factors may be of equal or greater importance. VL tends to affect poor populations with low nutritional status and in whom gastrointestinal and helminthic infections are common. Malnutrition can weaken both innate immunity and T cell functions (Anstead et al., 2001; Hughes and Kelly, 2006), and helminth exposures can shift the Th1/Th2 balance in favor of the *Leishmania* parasite (Hassan et al., 2006; O’Neal et al., 2007). Hormonal changes after puberty have also been suggested to influence susceptibility and young and adult men are over-represented among VL patients (Jeronimo et al., 2004; Nylen and Sacks, 2007). Experimental data on this aspect is limited, but in support of gender-associated differences in susceptibility, male hamsters are more susceptible to *L. donovani* infection (Anuradha et al., 1990) and testosterone treatment of macrophages has been found to enhance *L. donovani* replication (Liu et al., 2006).

## ANTIBODIES – FRIENDS OR FOES?

The role of the anti-leishmanial antibody response seen in VL patients is unclear. Patients with active VL have high level of anti-leishmanial IgE, IgM, and IgG (Ghosh et al., 1995; Anam et al., 1999; da Matta et al., 2000; Ryan et al., 2002). Moreover, people living in endemic region are regularly bitten by *Phlebotomus argentipes* and develop anti sand fly**salivary antibodies (Barral et al., 2000) indicating that the sand fly saliva antibody response could be used as a tool for evaluating exposure and/or risk of infection in endemic regions (Clements et al., 2010; Gidwani et al., 2011c).

It has been suggested that presences of anti-leishmanial antibodies could be predictive of disease (Singh et al., 2002), but this has not been confirmed. In a longitudinal serological study from an endemic area of Bihar state, India, where 33% of individuals were serological positive, only 3.5% of the seropositive individual converted to disease. The conversion rate in the seronegative group was not different (2.6%) during the 2 years of follow-up, implying that serological status in healthy individuals cannot predict the disease conversion (Gidwani et al., 2009; Ostyn et al., 2011).

While still limited in ability to distinguish between active and past or subclinical infection, antibodies have proven useful in diagnosis of VL disease (Sundar et al., 2002a; Sundar and Rai, 2002; Clements et al., 2010; Gidwani et al., 2011c). Apart from this, B cells and antibodies have historically not been considered to be of much importance in *Leishmania *infection. High levels of *Leishmania*-specific antibodies are observed in patients with VL, whereas CL patients lack *Leishmania*-specific antibodies or mount a very weak response. Thus, development of a strong humoral response is more associated with pathology than protection or resolution of disease (Galvao-Castro et al., 1984).

In mice, there are accumulating evidences that B cells and antibodies contribute to the VL pathology (Ronet et al., 2008; Deak et al., 2010). Mice lacking B cells were found to be less susceptible to *L. donovani* infection (Smelt et al., 2000) and in cutaneous models of leishmaniasis, immune complex formation and engagement of Fc-receptors have been found to promote parasite replication by driving IL-10 production in macrophages (Kane and Mosser, 2001; Buxbaum and Scott, 2005). IL-10 promotes the B cells survivals and plasma cells proliferation and also IgG isotype switch toward IgG1 and IgG3 (Caldas et al., 2005). Thus, high antibody titers and immune complex formation in VL may contribute to the elevated IL-10 levels observed and participate in the progressive decline in the immune status of VL patients.

However, a protective role of antibodies cannot be excluded. There is a high prevalence of seropositive healthy individuals in areas endemic for VL (Costa et al., 2002) and antibodies against *Leishmania* persist for a long time (15 years or more) after cure and presumed immunity to VL (Gidwani et al., 2011b). Presence of B cells and uptake of promastigotes through FcγRI on DC have, in mCL, been show to facilitate generation of protective T cell immunity (Scott et al., 1986; Woelbing et al., 2006). If this holds true in experimental VL is yet to be demonstrated. The requirement for B cell is not corroborated by the above-mentioned study by Smelt et al. (2000) showing that mice lacking B cells are highly resistant to experimental VL, nor supported by the recent study by Bankoti et al. (2012) suggesting that marginal zone B cells suppress protective T cells responses in the early stages of experimental VL.

## PROTECTIVE TH17 CELLS?

A protective role for Th17 cells in human VL was suggested by a recent longitudinal study carried out in the Sudan, showing correlation between the presence of *L. donovani*-specific T cells, secreting IL-17 and IL-22, and protection against developing VL (Pitta et al., 2009).

Th17 cells are primarily pro-inflammatory CD4 T cells that have the potential to secrete the prototypic cytokine IL-17 and express the transcription factors: related orphan receptor (ROR)γT and signal and transduction activator of transcription (STAT)-3. IL-17 act as a classic effector of innate immunity and induces expression of many innate inflammatory mediators, including IL-6, acute phase proteins, granulocyte colony stimulating factor (G-CSF), and prostaglandin E2, as such Th17 cells have been implicated in a number of immune-mediated disorders (reviewed in Tesmer et al., 2008). Activation of Th17 cells is intimately associated with recruitment of neutrophils, which may contribute to both protective and damaging aspects of Th17 cells. In human muco-cutaneous leishmaniasis there are implications of Th17 cells and neutrophils in pathological responses and destruction of tissue (Boaventura et al., 2010).

**FIGURE 1:**
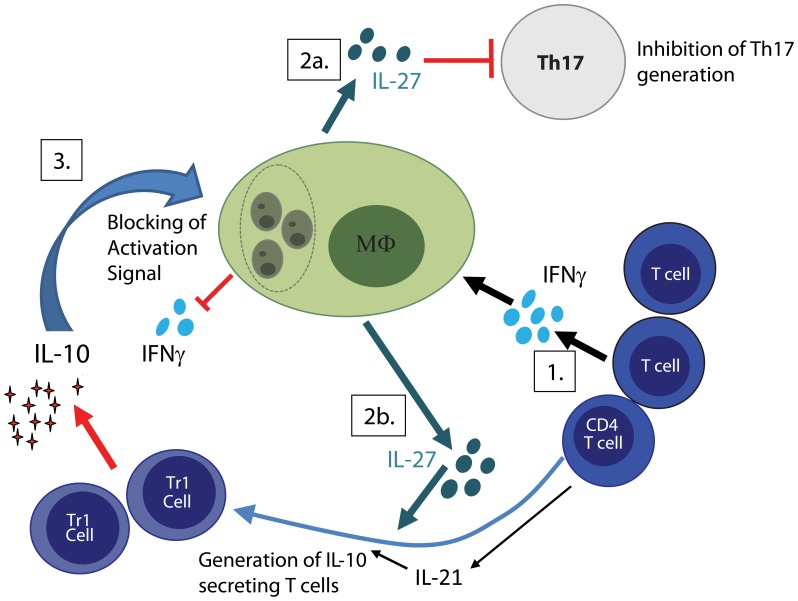
**Regulatory feedback loop facilitate parasite persistence**. (1) Activated T cells both CD4 and CD8 produce IFNγ, which in combination with IL-1β drives IL-27 production by macrophages. (2a) IL-27 inhibits generation of potentially protective Th17 cells and (2b) promotes together with IL-21 the generation IL-10 secreting T cells. (3) IL-10 cause down-regulation of IL-12, MHC class II as well as co-stimulatory molecules on macrophages and render macrophage unresponsive to activation signals such as IFNγ. Together this facilitates parasite replication and disease progression.

IL-27 is a cytokines central in regulation of Th17 cells, which mainly is produced by antigen-presenting cells. Studies in mice have demonstrated that IL-27 can inhibit the differentiation of Th17 cells involved in autoimmunity and pathogenic responses to infection (Batten et al., 2006; Stumhofer et al., 2006). In humans, IL-27 suppress IL-17 and IL-22 secretion by CD4 T cells cultured under Th17 polarizing conditions (Murugaiyan et al., 2009). In a recent study of Indian VL it was shown that patients with on-going disease had elevated IL-27 serum levels and increased expression of IL-27 transcripts (EBI-3 and IL-27p28) in splenic aspirates, while IL-17/RORγT transcripts were expressed in scarcity in the splenic biopsies both pre- and post- treatment (Ansari et al., 2011). Experimentally, IL-27 was moreover found to regulate the differentiation and expansion of antigen-specific IL-10 producing T cells (Ansari et al., 2011). In light of the prospective study carried out in the Sudan, implicating the protective role for Th17 cells (Pitta et al., 2009), the low expression of Th17-associated cytokines and transcription factors in patients with active VL combined with up-regulation of IL-27 implicate a role for IL-27 in VL pathogenesis. We suggest that IL-27 promote the differentiation and expansion of antigen-specific, IL-10 producing T cells and inhibit the potentially protective Th17 lineage and thereby facilitate parasite survival (**Figure 1**). However, it cannot be excluded that the reduction of Th17 seen in VL is an attempt of the body to control the pathological effects of Th17 cells and the inflammatory response driven by the parasite.

## REGULATORY T CELLS FAVOR PARASITE PERSISTENCE IN VL

The regulatory T cells (Treg) are broadly divided into CD4^+^CD25^+^Foxp3^+^ natural Treg (nTreg) and CD4^+^CD25^–^Foxp^–^ adaptive Treg or Tr1 cells. In humans, this division becomes more difficult since Foxp3 expression is inducible (Roncador et al., 2005). In mice, nTregs are generated during thymic development, whereas adaptive Treg arises during an encounter with antigen in the periphery. Natural Treg constitute 5–10% of peripheral CD4 T cells in human and naive mice and suppress several potentially tissue damaging effects *in vivo*. The absence of Treg is associated with a number of auto-immune conditions (reviewed in Maloy and Powrie, 2001).

Natural Treg cells have been shown to produce high amount of IL-10 and/or TGFβ, which in mCL contributes to the inability to complete eliminate *L. major* from skin after clinical cure (Belkaid et al., 2002). In humans, accumulation of Foxp3^+^ cells have been found in the skin of *L. braziliensis* patients (Campanelli et al., 2006) and patients suffering from PKDL (Katara et al., 2011). In both human and murine VL there is little support for accumulation or extensive activation of nTreg. In our studies conducted on patients from Bihar, India we could not reveal a major role of natural FoxP3^+^ Treg cells in overt human VL disease (Nylen et al., 2007; Maurya et al., 2010). There was no accumulation of these cells in the VL spleen, nor were their frequency changed after the treatment. Furthermore, Foxp3^–^CD4^+^ cells were found to be the major source of elevated IL-10 mRNA in spleen of VL patient (Nylen et al., 2007), supporting findings in murine models of VL showing that IL-10 production by CD4^+^FoxP3^–^ cells strongly correlate with disease progression (Stager et al., 2006). Yet, other groups suggest that Foxp3^+^ cells accumulate at the site of infection and play a role in both murine and human VL (Rodrigues et al., 2009; Rai et al., 2012). Rai et al. (2012) argue that FoxP3^+^ T cells are an important source of IL-10 in human VL and that these cells suppress effector T cell activation. Rodrigues et al. (2009) found TGFβ producing FoxP3^+^ T cells accumulating at sites of infection in experimental murine VL. Noteworthy, in both these studies non-Foxp3^+^ cells were found to be a source of IL-10 and while FoxP3 cells were in higher frequency in the bone marrow of VL patients (as compared to their PBMC), no significant difference in frequency of FoxP3 cells were detected pre-treatment as compared to post-treatment in either organ (Rai et al., 2012).

We believe, and most scientific data point to, that parasite driven adaptive Treg or Tr1 cells are more important than nTreg cells in suppressing anti-leishmanial immunity in VL.

## CD8 T CELLS IN VL

CD8 T cells have the capacity to directly lyse pathogen-infested cells and can produce high levels of the macrophage activating cytokine IFNγ. Like CD4 cells, CD8 cells subsets have regulatory capacity, which can serve to control tissue damages, but in that capacity may also promote parasite replication. As such, CD8 T cells may contribute to both cure and pathology in VL. In cutaneous forms of leishmaniasis CD8 cells have been associated with both tissue damage, as seen in muco-cutaneous lesions (Brodskyn et al., 1997; Tuon et al., 2008) and by cure, seen as an increase in the ability of CD8 cell from cutaneous *L. braziliensis *patients**to**respond to *Leishmania* *in vitro *(Da-Cruz et al., 1994; Coutinho et al., 1998). A lower CD4/CD8 ratio has been described in VL PBMC (Rohtagi et al., 1996; Taher et al., 2009). The alterations in blood cannot simply be explained by a preferential recruitment of CD4 T cells to the spleen since, in the early stages of human disease, VL is associated with a splenic influx of both CD4 and CD8 T cells (Nylen et al., 2007), implicating that CD8 cells may survive better than CD4 cells in VL patients. In the end stage of disease leukocytes are scanty and plasma cells and macrophages predominate in the spleen (Veress et al., 1977).

Experimental mouse models of VL show that CD8 T cells are important in control of *L. donovani*/*L. infantum* infection in the liver, through their ability to produce IFNγ and/or their cytolytic activity (Stern et al., 1988; Tsagozis et al., 2003, 2005; Polley et al., 2006). Moreover, CD8 T cells are, together with CD4 cells, required to control and prevent reactivation of VL in mice (Murray, 2005). CD8 T cell responses also provide a good correlate of immunity following vaccination (Stager et al., 2000).

CD8 T cells activated during *Leishmania *infections may not all be parasite-specific, as uninfected DCs that have matured during inflammation, can stimulate CD8 T cells to proliferate without the expression of their cognate ligands (Maroof et al., 2009). The role of these non-leishmanial-specific CD8 T cells in the infection is not clear. It has been shown that pre-existing memory CD8 T cells are expanded during *L. donovani* infection, and that they can provide increased resistance to previously encountered pathogens (Polley et al., 2005). However, bystander activated CD8 T cells cannot confer protection against VL, as only CD8 cell recognizing cognate antigen were found to be protective when given as immunotherapy (Polley et al., 2006).

*Leishmania donovani* seem to be able to elude the expansion of parasite-specific CD8 T cells by causing exhaustion and death of CD8 T cell (Joshi et al., 2009). Blockade of B7-H1, the ligand for the inhibitory receptor PD-1, was found to increase survival of CD8 cells and induce protective immunity, suggesting that blockade of inhibitor receptors as immune therapy in VL (Joshi et al., 2009). Although, the parasite have means to limit T cells response in murine VL, the mice ultimately control the disease and CD8 cells are important in the hepatic control of parasite propagation. In mice, formation of granulomas is central in control of the parasite in the liver. Kupfer cells infiltrating the granuloma have the ability to harbor amastigotes and present antigen (You et al., 2008; Beattie et al., 2010). *In vivo* imaging has revealed that these infected Kupfer cells interact closely with antigen-specific CD8 T cells and facilitate accumulation of effector CD8 T cells within the liver granuloma (Beattie et al., 2010). CD8 T cells are not well described in human VL and their functions are at large unknown.

## IMMUNOTHERAPY TO IMPROVE TREATMENT EFFICACY

Despite significant advances in the treatment of VL, conventional chemotherapy is associated with toxicity and relapse of disease. Immunotherapeutic strategies, aimed to strengthen *Leishmania-*specific immune response, prior to or in synergy with conventional therapy, may lower the required dose or treatment regimen and thus toxicity. Immunotherapy may also improve the drug efficacy, reduce emergence of drug resistant strains, and circumvent the problems of treatment in immuno-compromised hosts.

Attempts to identify cytokines, which may be useful in VL therapy, have focused on cytokines thought to act on the balance between Th1 and Th2 cells. This despite the lack of evidence for a clear role for Th2 cells in pathology of symptomatic human VL (Carvalho et al., 1994; Ghalib et al., 1995).

The combination of IFNγ and SbV therapy has shown beneficial effects in treatment of patients with VL and diffuse cutaneous leishmaniasis (DCL; Badaro and Johnson, 1993). However, targeting regulatory pathways and cytokines may prove more efficient than boosting effector cytokines. Clinical and experimental data suggest IL-10 as a key target for VL immunotherapy. By inhibiting IL-10, the balance between effector and regulatory cytokines may be shifted. IL-10 blockade may enhance the function of antigen-presenting cells and moreover increase the ability of infected macrophage to respond appropriately to IFNγ, thus promoting the killing of parasite, as shown *in vitro* (Nylen et al., 2007; Gautam et al., 2011). This may reduce the dose and/or length of treatment the patients receive. Neutralization of IL-10 has been tested in patients with systemic lupus erythematosus (SLE), an immune complex disorder. In these patients anti-IL-10 treatment decreased disease activity and restored impaired T lymphocyte function (Llorente et al., 2000). As stated above, in *ex vivo* cultures of splenic aspirates from patients with VL, the presence of anti-IL-10 antibody was shown to promote the killing of parasite and significantly increased IFNγ and TNFα secretion by spleen cells (Gautam et al., 2011). These findings clearly demonstrate the potential benefits of IL-10 inhibition as a therapeutic approach in human VL. Other potential targets could be receptors expressed on Treg or their corresponding ligands on effectors cells, such as PD-1, it’s ligands PD1-L (B7-H1) or CTLA-4. Targeting these regulatory pathways has proven effective in experimental VL (Murray et al., 2003a; Joshi et al., 2009). If these molecules are implicated in human VL and could serve as putative targets remain to be demonstrated.

## CONCLUDING REMARKS

Failure to eliminate or control parasite replication results in continuous parasite driven immune activation and production of pro-inflammatory molecules. These pro-inflammatory molecules will, if not controlled, cause tissue damage and need to be tightly regulated. As a self-regulating negative feedback mechanism many pro-inflammatory molecules induce regulatory responses. We envisage that parasite induced IFNγ, in combination with IL-1β, act on DCs and macrophages to promote production of IL-27. IL-27, in turn, block generation of potentially protective Th17 cells and facilitates generation of IL-10 producing T cells (depicted in **Figure 1**). IL-10 effectively down-regulates the antigen-presenting capacity both of DC and macrophages and impairs the ability of macrophages to respond to activation signals. While the generation of regulatory response temporarily reduces tissue damage, they simultaneously allow parasite propagation. Parasite persistence result in a continued pro-inflammatory response creating a loop that causes exhaustion of the immune system and failure to generated appropriate CD4 T cell responses. By temporarily breaking the regulatory feedback loop, using immune modulatory therapies to treat VL, the balance may be shifted allowing macrophage activation, killing of the parasite and generation of curing and protective CD4 T cell response.

## Conflict of Interest Statement

The authors declare that the research was conducted in the absence of any commercial or financial relationships that could be construed as a potential conflict of interest.
